# Supplementary material for: Cross-stress gene expression atlas of Marchantia polymorpha reveals the hierarchy and regulatory principles of abiotic stress responses
Source: Nat Commun. 2023 Feb 22;14:986. doi: 10.1038/s41467-023-36517-w (PMC9946954; doi:10.1038/s41467-023-36517-w)
Supplement: Supplementary file 3 — Description of Additional Supplementary Files [file 41467_2023_36517_MOESM3_ESM.pdf]

Description of Additional Supplementary Files:

Supplementary Data 1: Summary of experiment labels to experiment conditions and mapping statistics from Kallisto.

Supplementary Data 2: TPM normalized gene expression matrix for Marchantia experiments

Supplementary Data 3: Raw gene expression matrix for Marchantia experiments

Supplementary Data 4: PCC values within Marchantia experiment replicates

Supplementary Data 5: Summary of DESeq2 output of Marchantia stress experiments

Supplementary Data 6: List of genes that are identified to be differentially expressed in both controls with corresponding MapMan bins and annotation. 'UP' and 'DOWN' in L2FC_D2 and L2FC_H2 columns indicate significant up and downregulation when compared against control D2 and H2 respectively. Controls D2 and H2 refer to Controls from batches L and F in supp. data 1 respectively.

Supplementary Data 7: List of Marchantia transcription factors, their robust response in specific stresses and functional studies found during a literature search.

Supplementary Data 8: Evidence of function in Arabidopsis orthologs of Marchantia transcription factors based on literature and gene expression changes.

Supplementary Data 9: Incidents of evidence in Arabidopsis (yellow) for each group of robustly responding Marchantia transcription factor (green)

Supplementary Data 10: Cold-Nitrogen deficiency specific genes as obtained from conekt.plant.tools
