# Supplementary material for: Cross-stress gene expression atlas of Marchantia polymorpha reveals the hierarchy and regulatory principles of abiotic stress responses
Source: Nat Commun. 2023 Feb 22;14:986. doi: 10.1038/s41467-023-36517-w (PMC9946954; doi:10.1038/s41467-023-36517-w)

Heat stress

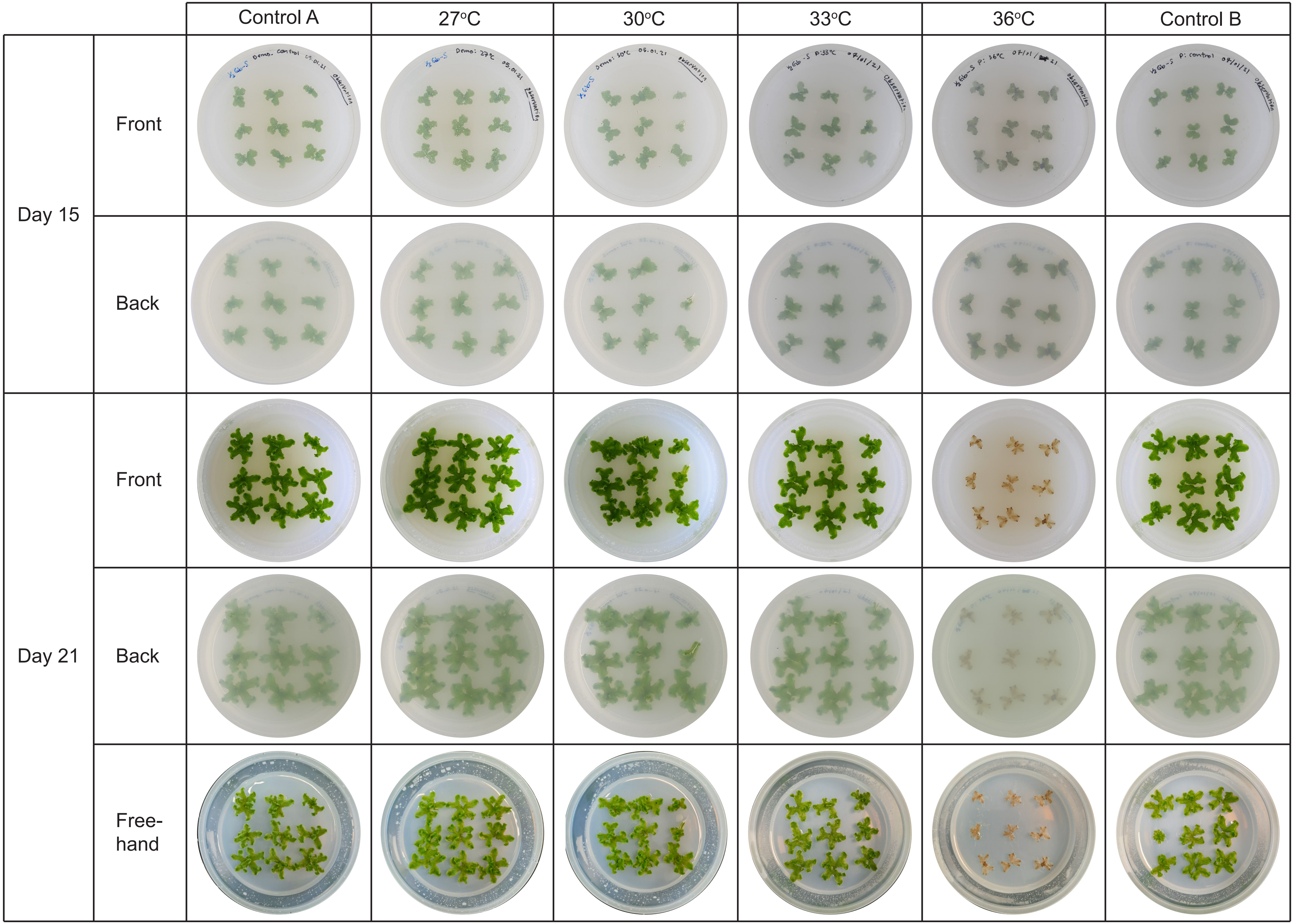
Control A
27°C
30°C
33°C
36°C
Control B
Day 15
Front
Back
Day 21
Front
Back
Free-hand

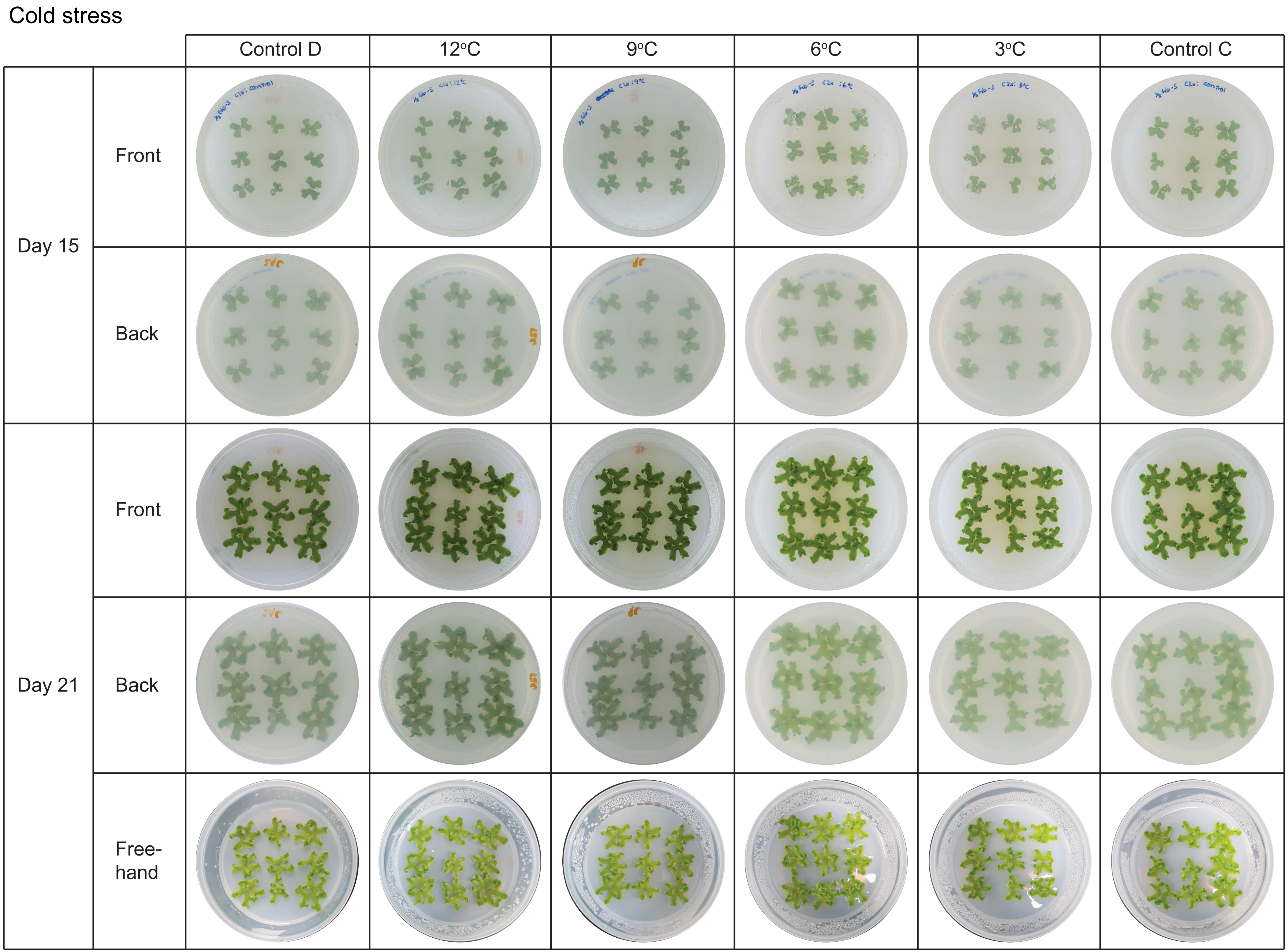
Cold stress
Control D
12°C
9°C
6°C
3°C
Control C
Day 15
Front
Back
Day 21
Front
Back
Free-hand

Osmotic stress 1

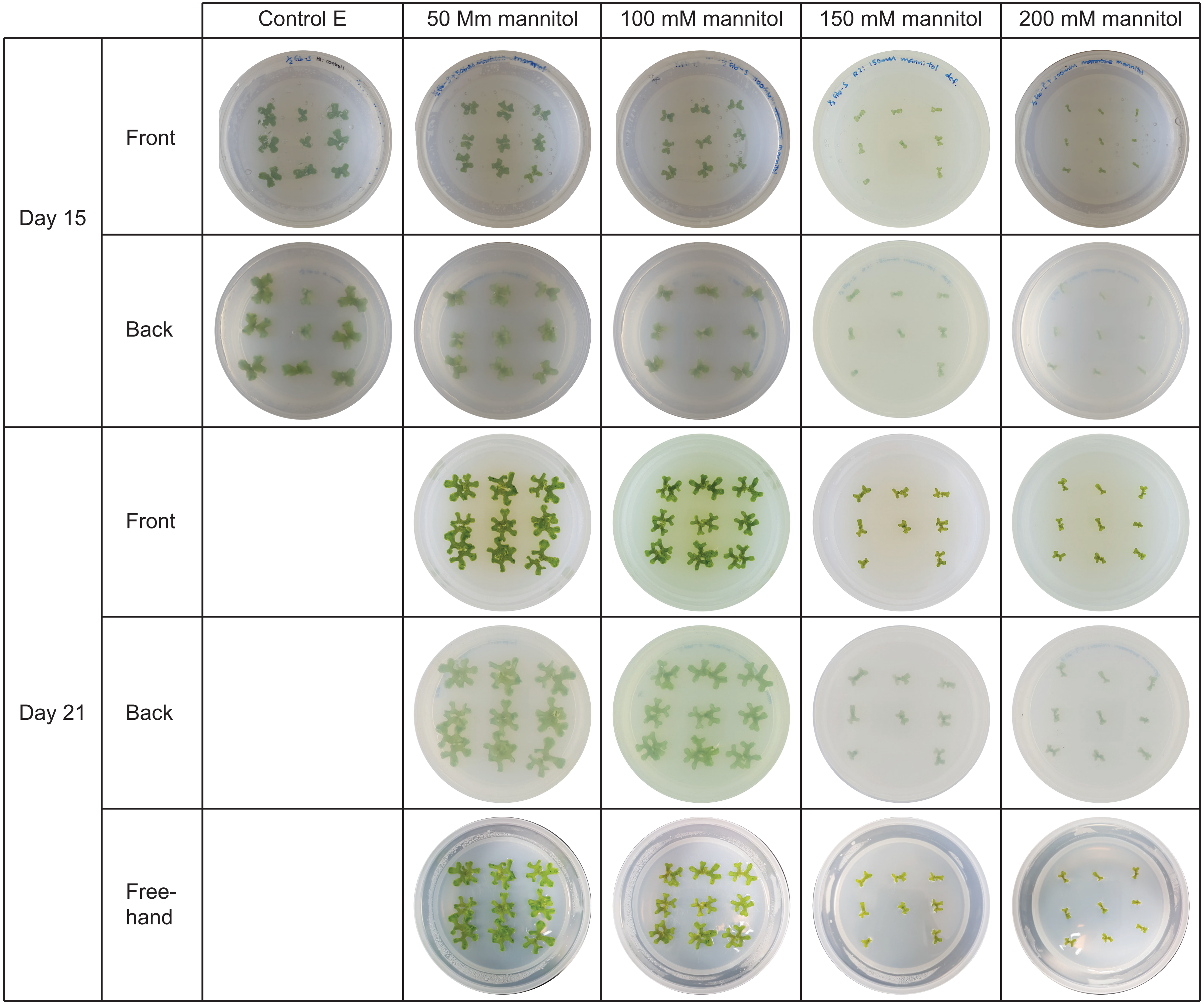

Osmotic stress 2

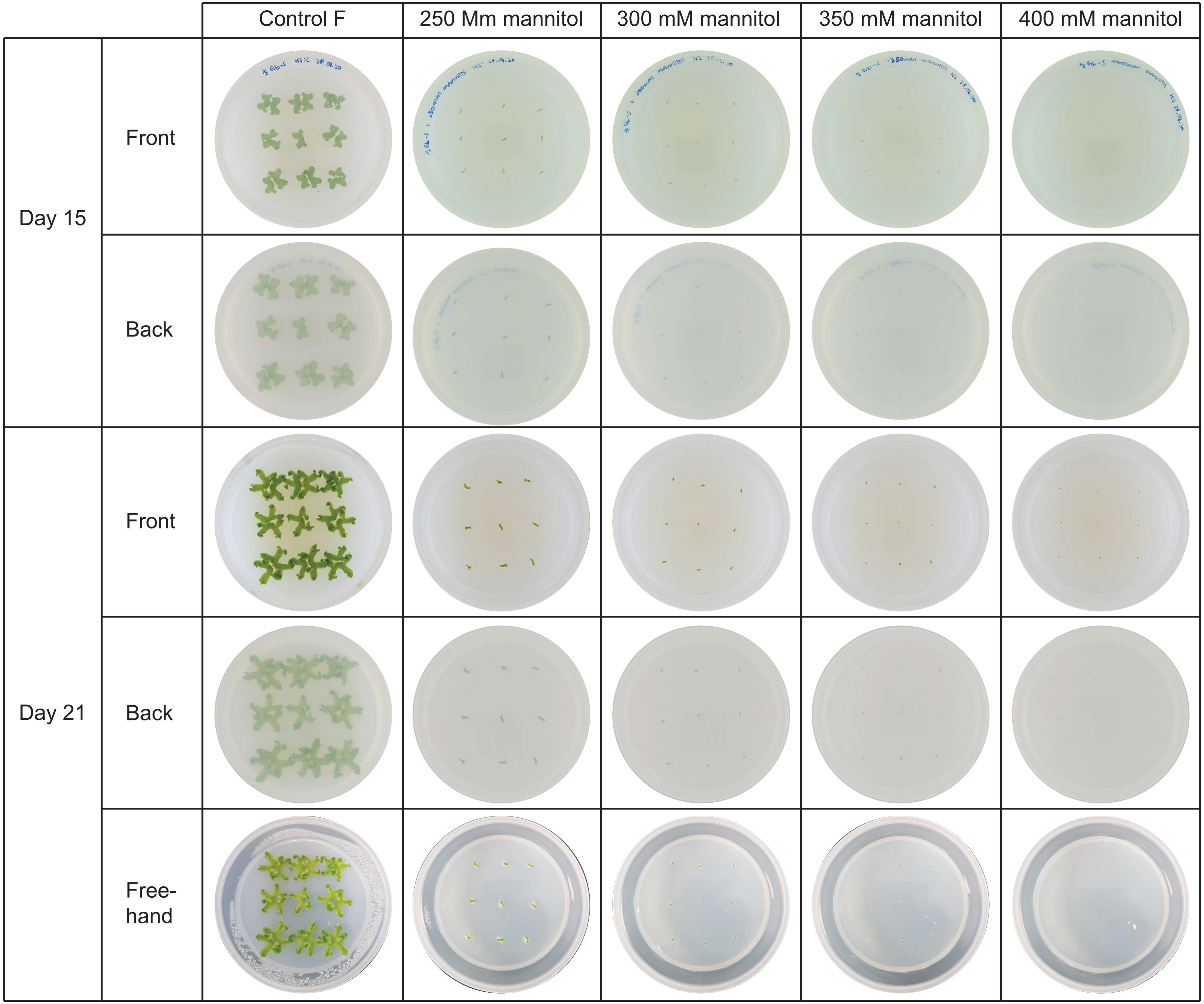

Salt stress 1

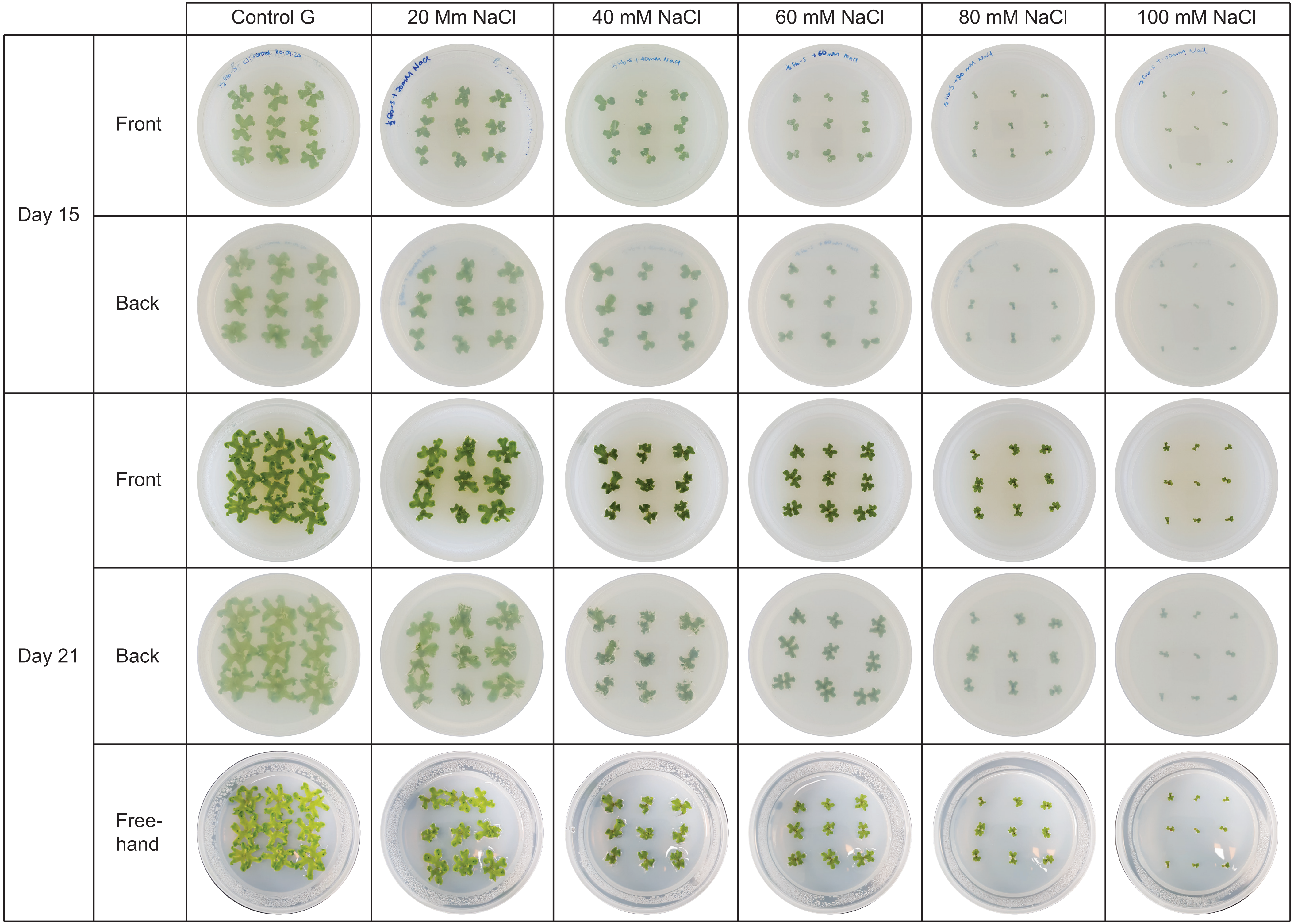

Salt stress 2

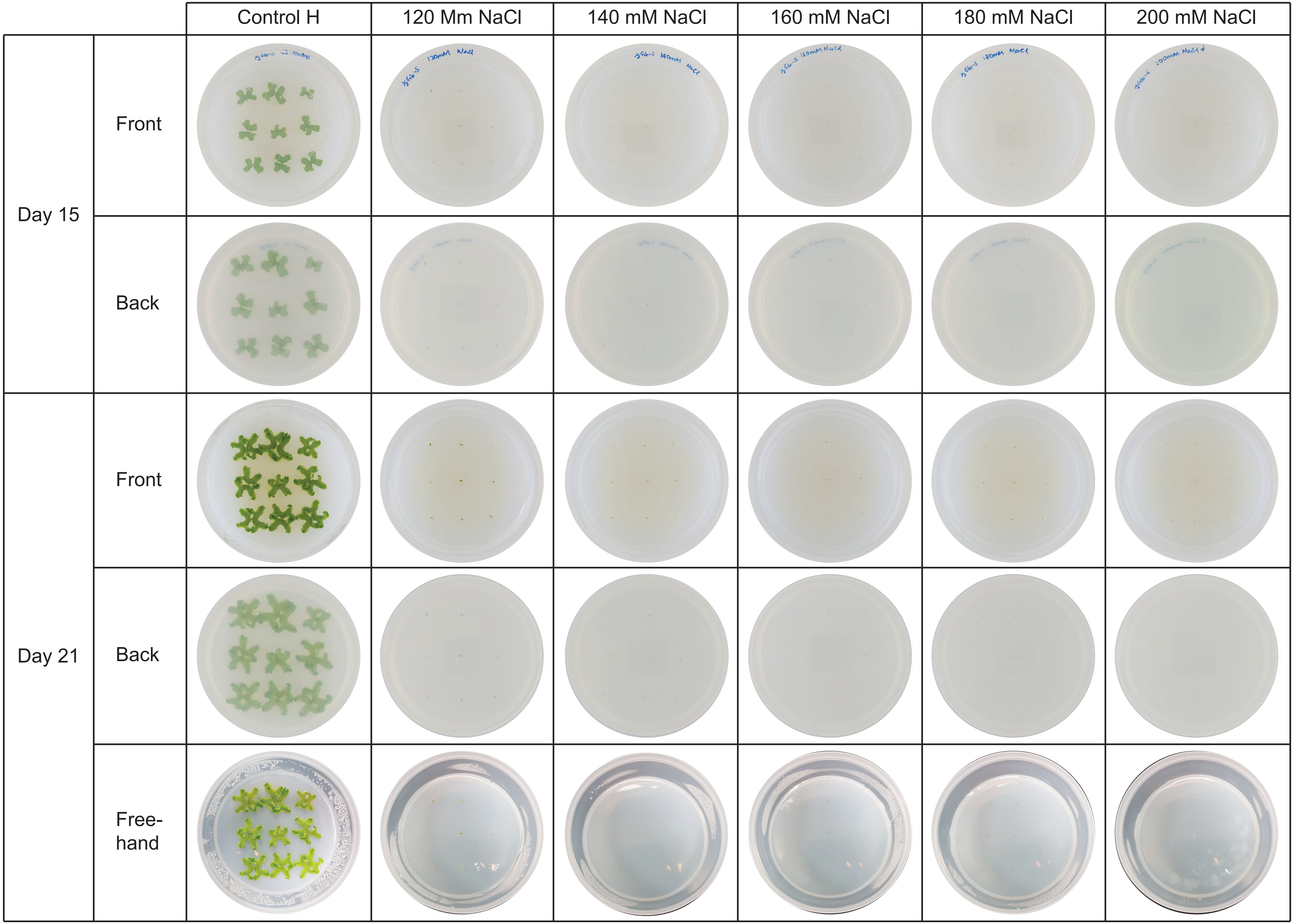

Light stress 1

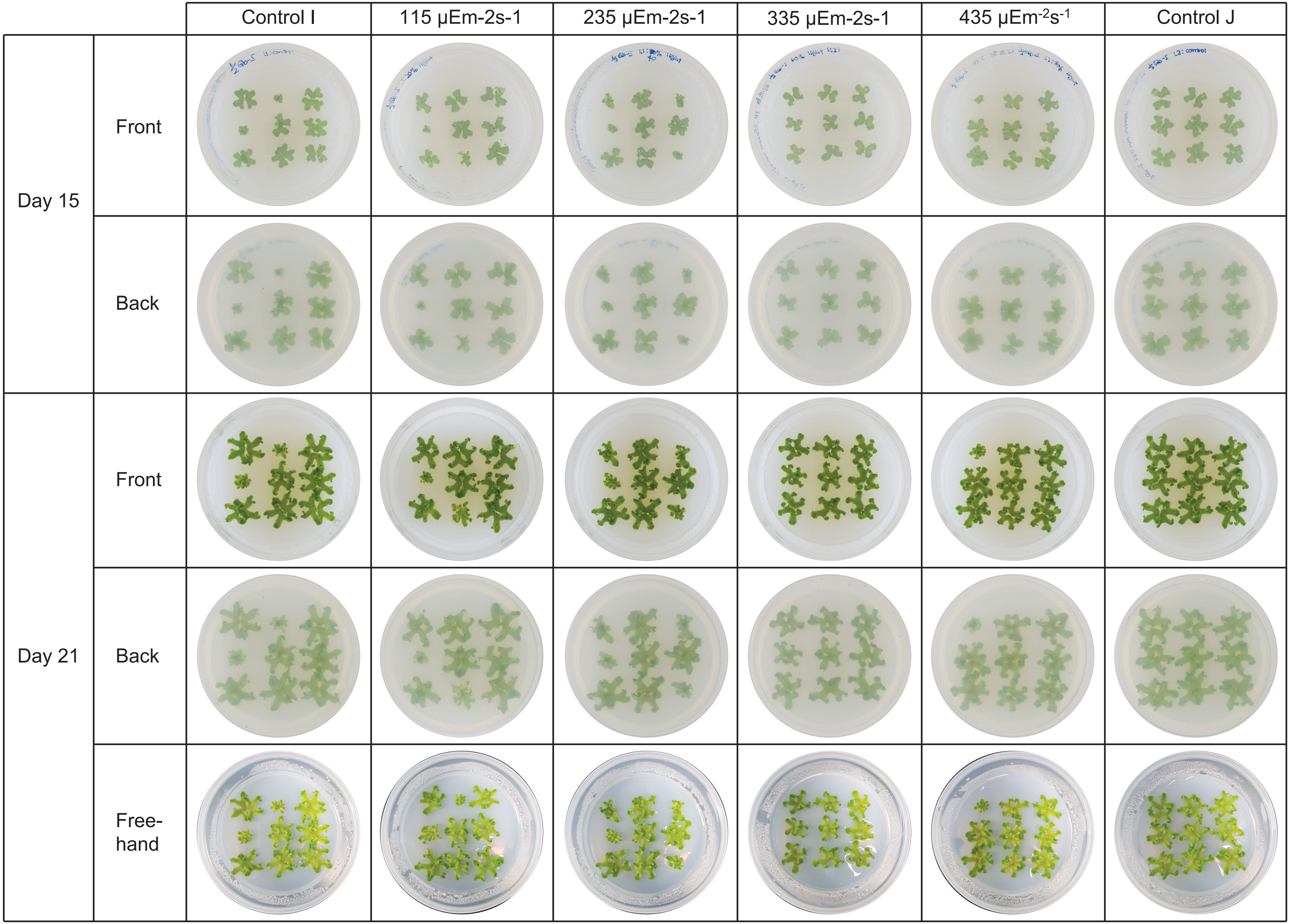

Light stress 2

		Control K	535 µEm-2s-1
Day 15	Front	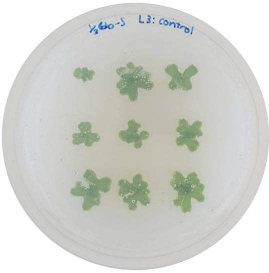	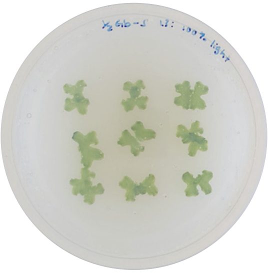
	Back	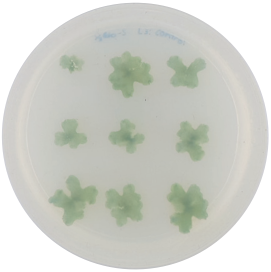	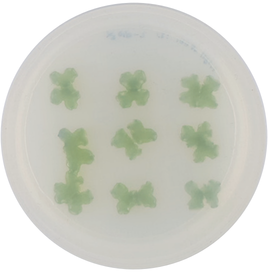
Day 21	Front	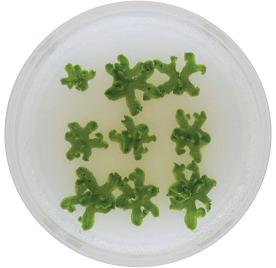	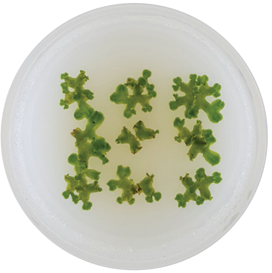
	Back	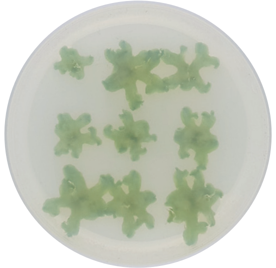	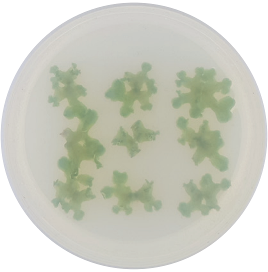
	Free-hand	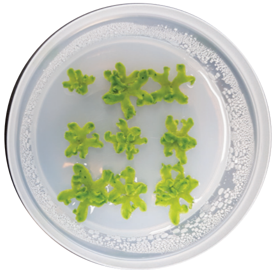	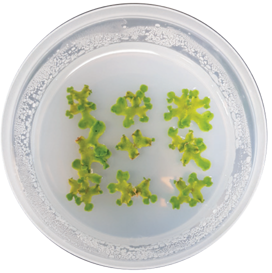

Dark stress 1

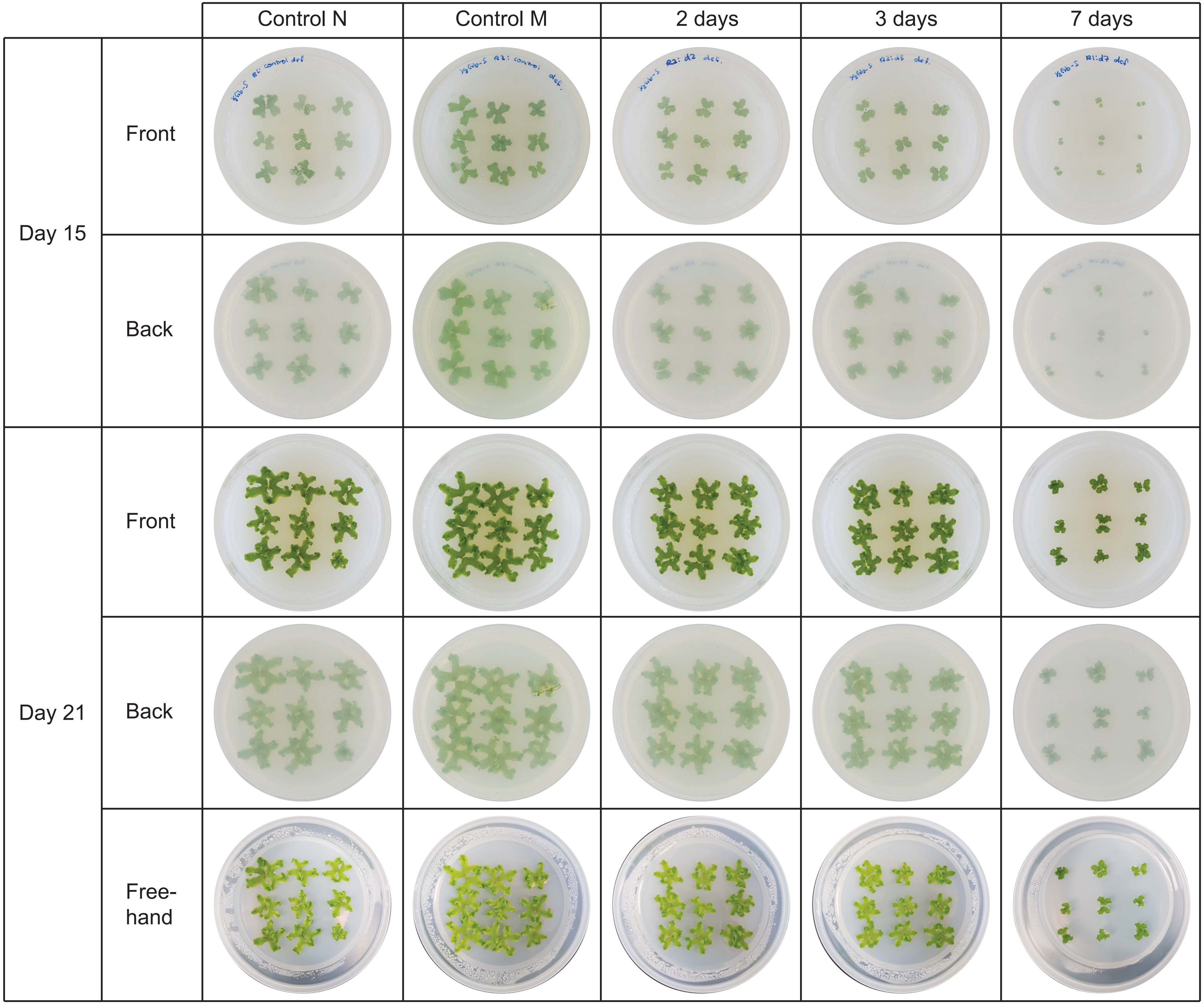

Dark stress 2

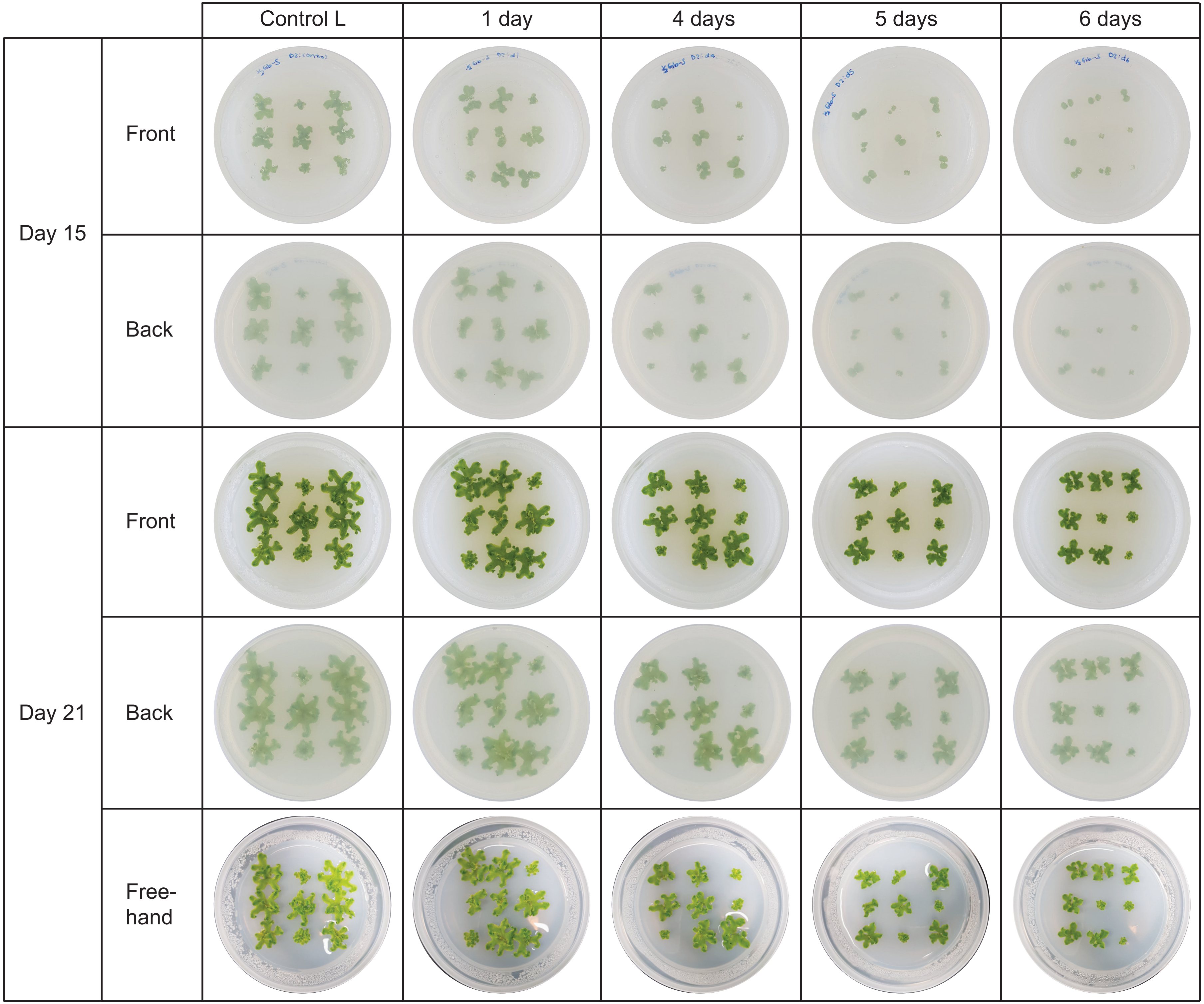

Nitrogen stress 1

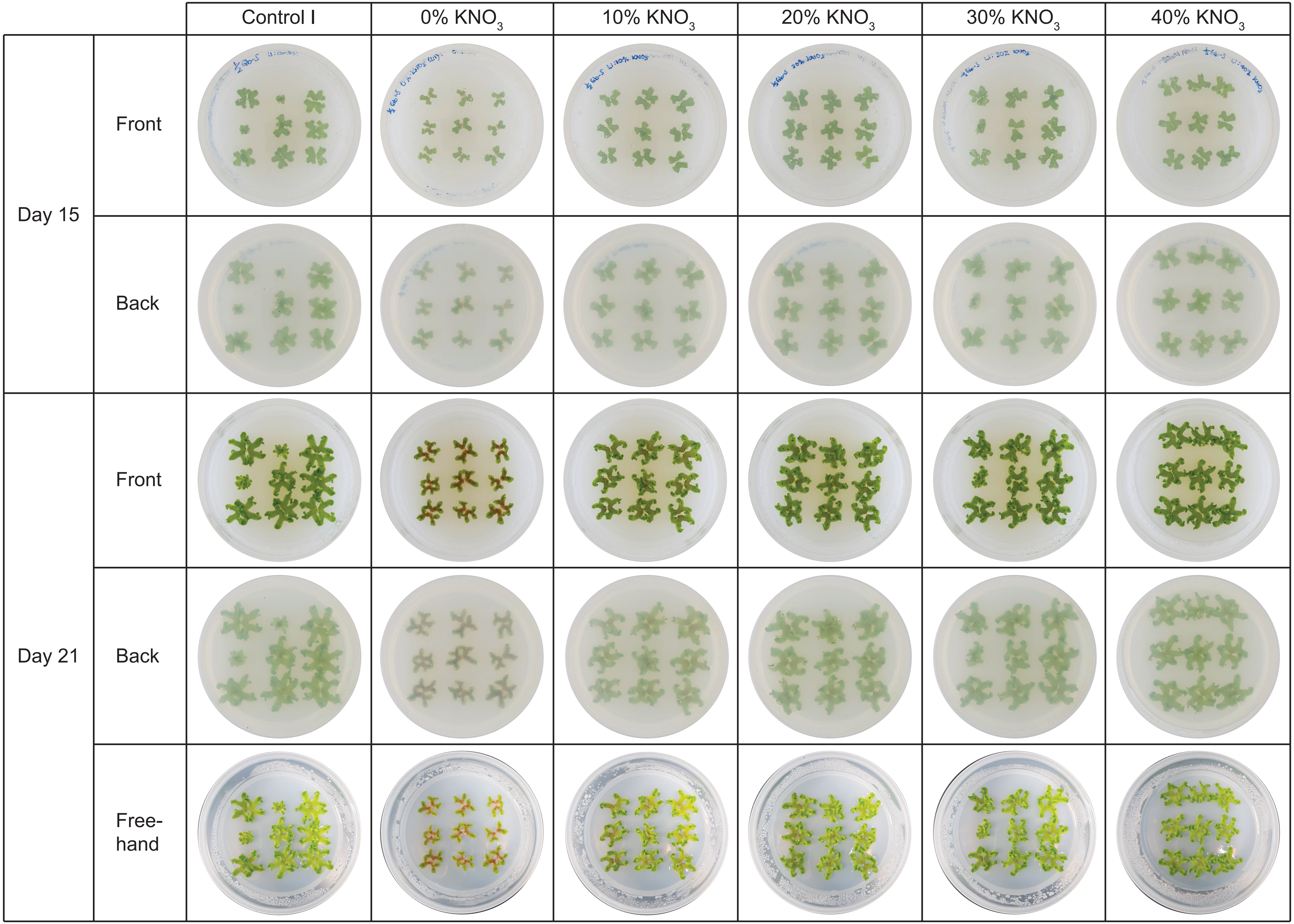

Nitrogen stress 2

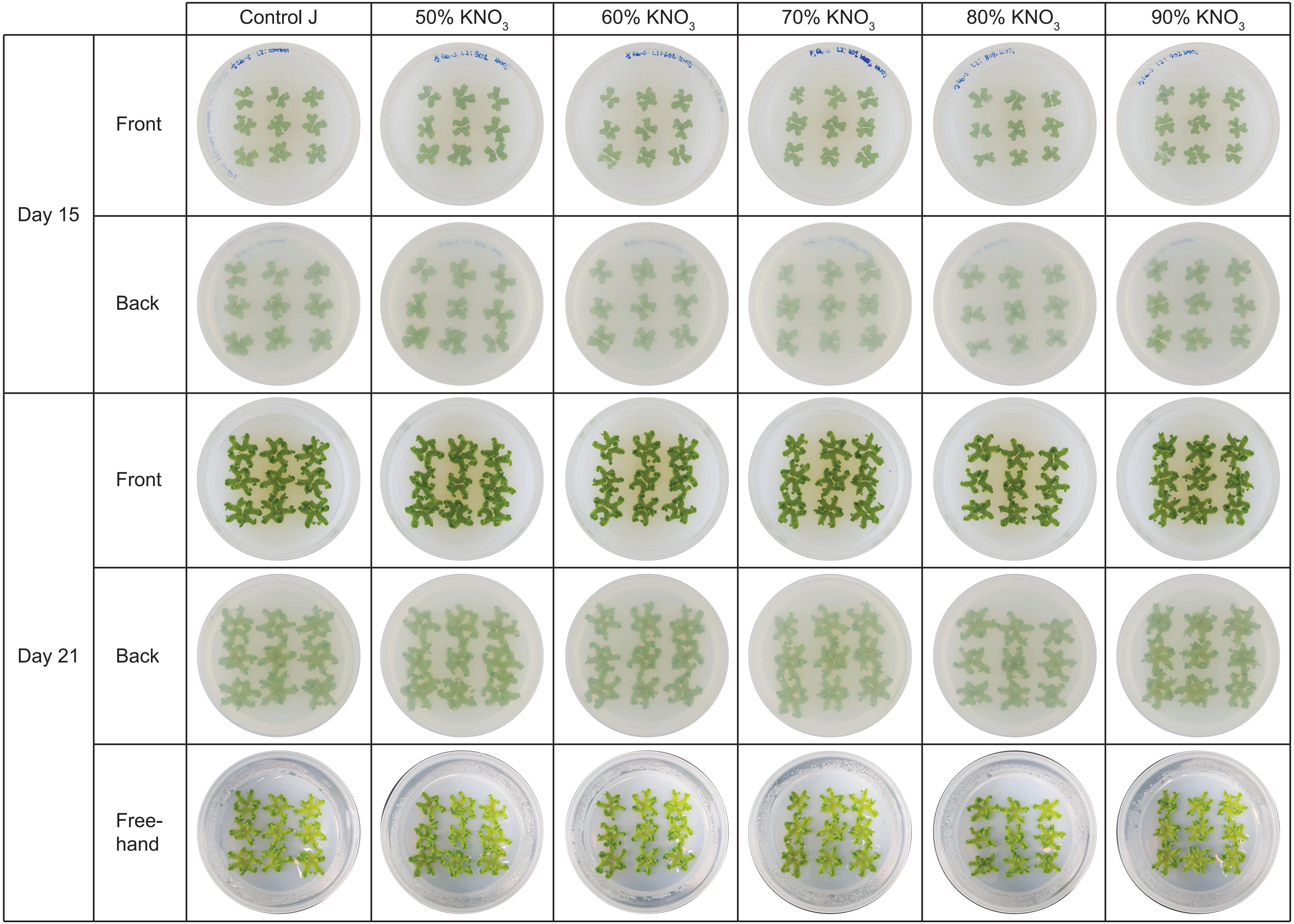

Supplement: Supplementary file 15 — Source Data [file 41467_2023_36517_MOESM15_ESM.zip › source data part 1.pdf]
